# Compromised Conscience: A Scoping Review of Moral Injury Among Firefighters, Paramedics, and Police Officers

**DOI:** 10.3389/fpsyg.2021.639781

**Published:** 2021-03-31

**Authors:** Liana M. Lentz, Lorraine Smith-MacDonald, David Malloy, R. Nicholas Carleton, Suzette Brémault-Phillips

**Affiliations:** ^1^Faculty of Health Science, Western University, London, ON, Canada; ^2^Canadian Institute for Public Safety Research and Treatment, University of Regina, Regina, SK, Canada; ^3^Heroes in Mind Advocacy and Research Consortium, Faculty of Rehabilitation Medicine, University of Alberta, Edmonton, AB, Canada; ^4^King’s University College, University of Western Ontario, London, ON, Canada

**Keywords:** firefighter, paramedic, singular, police officer, moral injury, moral distress, review

## Abstract

**Background:**

Public Safety Personnel (e.g., firefighters, paramedics, and police officers) are routinely exposed to human suffering and need to make quick, morally challenging decisions. Such decisions can affect their psychological wellbeing. Participating in or observing an event or situation that conflicts with personal values can potentially lead to the development of moral injury. Common stressors associated with moral injury include betrayal, inability to prevent death or harm, and ethical dilemmas. Potentially psychologically traumatic event exposures and post-traumatic stress disorder can be comorbid with moral injury; however, moral injury extends beyond fear to include spiritual, cognitive, emotional or existential struggles, which can produce feelings of severe shame, guilt, and anger.

**Objective:**

This scoping review was designed to identify the extant empirical research regarding the construct of moral injury, its associated constructs, and how it relates to moral distress in firefighters, paramedics, and police officers.

**Methods:**

A systematic literature search of peer-reviewed research was conducted using databases MEDLINE, EMBASE, APA PsychInfo, CINHAL PLUS, Web of Science, SCOPUS, and Google Scholar. Included studies were selected based on the inclusion criteria before being manually extracted and independently screened by two reviewers.

**Results:**

The initial database search returned 777 articles, 506 of which remained after removal of duplicates. Following review of titles, abstracts, and full texts, 32 studies were included in the current review. Participants in the articles were primarily police officers, with fewer articles focusing on paramedics and firefighters. There were two studies that included mixed populations (i.e., one study with police officers, firefighters, and other emergency service workers; one study with paramedic and firefighter incident commanders). Most studies were qualitative and focused on four topics: values, ethical decision-making, organizational betrayal, and spirituality.

**Conclusion:**

Public safety organizations appear to recognize the experience of moral distress or moral injury among public safety personnel that results from disconnects between personal core values, formal and informal organizational values, vocational duties, and expectations. Further research is needed to better understand moral distress or moral injury specific to public safety personnel and inform training and treatment in support of public safety personnel mental health.

## Introduction

Public safety personnel (PSP; e.g., border services officers, public safety communications officials, correctional workers, firefighters paramedics, police, (Canadian Institute of Public Safety Research and Training [CIPSRT], 2019) work in fast-moving and unpredictable environments (Angehrn et al., 2020) that typically involve exposures to potentially psychologically traumatic events (Corneil et al., 1999; Carleton et al., 2019). PSP are often required to make quick decisions and act urgently to protect both the public and themselves; accordingly, PSP work in professions that intrinsically involve a moral endeavor focused on an ethic of care (Papazoglou, 2013; Qashu Lim, 2017). The duties performed by PSP occur in complex contexts where ethical practice happens in a social order within a value framework (Norberg, 2013). PSP have multiple responsibilities including patient advocacy, social services, enforcement, protection, and community partnerships. PSP are also part of professional and bureaucratic systems wherein codes of conduct, explicit and implicit duties, and standards of practice, including the law, must figure into their decision-making behavior (i.e., an ethic of duty) (Qashu Lim, 2017). PSP often find themselves in uncertain or ambiguous and potentially traumatic circumstances where resolution may require rapidly acting against the interests of at least one person (Herbert, 1996; Angehrn et al., 2020). Accordingly, their decisions may have serious implications that can negatively impact the mental health of PSP, particularly if errors are made or decisions contradict their personal values.

Public safety personnel report higher levels of mental disorder symptoms than the general public (Carleton et al., 2018; Wagner et al., 2020), which appears related to potentially psychologically traumatic events (Carleton et al., 2019) and occupational stressors (Carleton et al., 2020). Such disorders are increasingly referred to as either operational stress injuries or posttraumatic stress injuries (PTSI; Canadian Institute of Public Safety Research and Training [CIPSRT], 2019). The historical focus on exposure to potentially psychologically traumatic events as a key mechanism of occupational stress injuries is consistent with the available research on posttraumatic stress disorder (Maddox et al., 2019); however, a diversity of interacting mechanisms as important for mental health (Acquadro Maran et al., 2018). The recent evidence suggests that stressors specific to the organization (e.g., feeling that different rules apply to different people) or the operations (e.g., negative comments from the public) contribute to occupational stress injuries (Harrison, 2019; Carleton et al., 2020). For example, PSP often find themselves in impossible situations while attempting to navigate to moral safety amidst their dual ethical obligations for care and duty. The impact of such events can occur alongside deep philosophical harm (i.e., existential threat to one’s sense of self) for PSP who experience conflicting values or direct challenges to their morality. The conflicts and challenges can potentiate moral dilemmas and moral frustrations that lead to distress and impairment that may be referred to as moral injury. The research on moral injury has focused almost exclusively on military contexts, but PSP also encounter challenging potentially psychologically traumatic events (Corneil et al., 1999; Papazoglou, 2013; Carleton et al., 2019) that may result in a PTSI that includes moral injury.

### Moral Injury

The construct of moral injury has historical roots from spiritual, religious, and philosophical traditions, as well as from the history of attempts to manage potentially traumatic exposures (Tick, 2012). Early psychoanalytic work with Vietnam veterans implicated the experience of an “undoing of character” or “selfhood” stemming from deeply embedded moral woundedness as a critical component of PTSI (Shay, 1994, 2014). Moral injury has more recently been defined as a “… particular trauma syndrome including psychological, existential, behavioral, and interpersonal issues that emerge following perceived violations of deep moral beliefs by oneself or trusted individuals” (Jinkerson, 2016). While the mechanisms underlying moral injury remain unknown, exposures to at least one potentially morally injurious event (PMIE) are believed to be a prerequisite (Griffin et al., 2019). In military contexts, PMIEs appear focused on “perpetrating, failing to prevent, bearing witness to, or learning about acts that transgress deeply held moral beliefs and expectations” (Litz et al., 2009). Military PMIEs include, but are not limited to, disproportionate killing or violence, harming civilians, the inability to act for the protection of women and children, moral compromise, personnel or organizational betrayals, and challenging homecomings (Drescher et al., 2011; Vargas et al., 2013; Currier et al., 2015; Schorr et al., 2018).

Moral injury has been associated with potentially psychologically traumatic events and PTSI (Nash et al., 2010; Jordan et al., 2017), including PTSD (Koenig et al., 2019; Papazoglou et al., 2020); nevertheless, moral injury is distinct. PTSD is associated with threat and fear-based mechanisms (Maddox et al., 2019), whereas moral injury does note require fear-based mechanisms or responses. Moral injury can involve cognitive, emotional, spiritual, or existential struggles (Buechner and Jinkerson, 2016; Barnes et al., 2019; Griffin et al., 2019). The symptoms of moral injury can impact psychological, emotional, social, and spiritual domains of health. Exposure to PMIEs may increase the risk for moral injury, as well as other PTSI (Battles et al., 2018; Currier et al., 2019; Koenig et al., 2019), and may be predictive of PTSD (Papazoglou et al., 2020). Over half of Canadian Armed Forces members deployed to Afghanistan reported at least one PMIE exposure, and members exposed to PMIE were significantly more likely to report past-year difficulties with PTSD and major depressive disorder (Nazarov et al., 2018). Preliminary research results suggest moral injury is also a strong predictor of suicide among military personnel and veterans (Bryan et al., 2014, 2018; Ames et al., 2019), possibly due to significant emotional dysregulation (e.g., feelings of shame, guilt, contempt, anger, disgust) (Williamson et al., 2018). Moral injury also appears to challenge a person’s sense of self and spirit, sense of trust, core beliefs, meaning, and purpose, as well as challenging fundamental relationships with self, others, and the sacred/transcendent (Currier et al., 2015; Carey et al., 2016; Smith-MacDonald et al., 2018).

### Moral Distress

Moral distress has been a concept within healthcare literature since the 1990s, which is much earlier than the more recent development of moral injury. The term “moral distress” was first coined by the nurse-philosopher, Jameton, (Jameton, 1984) and was defined as the negative experience “when one knows the right thing to do, but institutional constraints make it nearly impossible to pursue the right course of action” (p. 6). Moral distress has been widely studied in healthcare, predominantly with nurses and more recently doctors. A recent systematic review indicated several PMIEs relating to moral distress include: (1) organizational aspects including ethical climate (e.g., lack of support, lack of respect, lack of involvement in decision making), difficult nurse–physician collaboration, and job characteristics (e.g., workload, not enough time for patient-care); and (2) low levels of structural empowerment, psychological empowerment, autonomy, and poor access to occupational resources (e.g., not enough beds, medication, supplies) (Lamiani et al., 2017). Feelings of powerlessness regarding treatment decisions, high-intensity medical environments, lack of authority, and high responsibility may create an optimal environment for moral distress (Hefferman and Heilig, 1999). Limitations to moral agency may lead to moral distress due to a “lack of empowerment associated with the hierarchical nature of nursing, lack of time, a high workload, as well as the ‘politics of healthcare.’ These constraints show that the system and nurses may have diverging views regarding patient ontology” (Fortier and Malloy, 2019, p. 5).

Moral distress appears to be related to a myriad of reactions including anger, loneliness, depression, guilt, anxiety, feelings of powerlessness, and emotional withdrawal, all of which lead to related physical symptoms (Huffman and Rittenmeyer, 2012). Moral distress appears to negatively impact healthcare professionals’ professional attitudes, job satisfaction, and satisfaction with quality of care provided, and can lead to absenteeism, emotional withdrawal from patients, experiences of burnout and compassion fatigue, and leaving the profession (Ford, 2010; Oh and Gastmans, 2015; Fistein and Malloy, 2017). Indeed, a singular focus on potentially psychologically traumatic event exposures for frontline care may not adequately address the full scope of harm caused to frontline personnel.

The published research on moral distress and moral injury needs to be expanded to include other helping professions where people are exposed to potentially psychologically traumatic events and PMIE while performing work-related duties and tasks (e.g., firefighters, paramedics, police officers, and other PSP). Research on moral injury has been hindered by ambiguity regarding the relationship between moral distress and moral injury; specifically, whether moral distress and moral injury are identical or conceptually similar but ontologically different based on service environment (i.e., military vs. healthcare). Moral distress and moral injury could be conceptualized as constructs along a continuum wherein moral distress is less severe (i.e., emotional responses to relatively common moral dilemmas) than moral injury (i.e., distress culminates in symptoms that are problematic, impairing, and potentially pathological; (Farnsworth et al., 2017; Papazoglou and Chopko, 2017; Williamson et al., 2018). There are several theoretical publications examining moral injury in firefighter, paramedic, and police officer populations (Miller, 2007; Bremer and Sandman, 2011; Papazoglou and Chopko, 2017; Murray, 2019), but empirical research clarifying the relationship between moral distress and moral injury, and how those constructs may impact the prevalence and treatment of PTSI is lacking.

#### Purpose

The current scoping review was designed to identify extant empirical research regarding the construct of moral injury, associated constructs, and relationships to moral distress in firefighters, paramedics, and police officers.

## Materials and Methods

### Study Design

The main question of the current scoping review was: “What is the current state-of-evidence regarding moral injury and associated constructs in police officer, firefighter, and paramedic populations?” The review proceeded in five stages (Arksey and O’Malley, 2005): (1) Identifying the research question; (2) Identifying relevant studies; (3) Selecting studies; (4) Charting study data; and (5) Collating, summarizing, and reporting the results.

### Search Strategy

A research librarian supported a systematic literature search which was conducted on April 30, 2020 using databases including CINHAL PLUS, EMBASE, Google Scholar, MEDLINE, OVID SCOPUS, and Web of Science. The following search terms were developed and were used for the OVID PsycINFO database search on which the other searches were based. OVID PsycINFO was selected as the first database because it was perceived to potentially have the most related articles. An additional manual search was performed across the reference lists of selected articles that met the inclusion criteria. Search terms used in other databases can be found in Supplementary Appendix I.

TOPIC: ("moral^*^ injur^*^" or "moral repair" or "moral dilemma^*^" or "morals" or "moral distress" or guilt or shame or grief or "compassion fatigue" or betrayal or "sanctuary trauma" or "moral Suffering" or spirit^*^) AND TOPIC: (police^*^ or firefighter^*^ or firem^*^ or EMT or EMTs or Emergency medical technician^*^ or paramedic^*^ or "public safety personnel" or "first responder^*^" or "law enforcement" or "medic" or medics or ambulance)

### Inclusion Criteria

There were two independent reviewers who identified and selected studies for inclusion that examine foundational constructs of moral injury, potentially morally injurious experience, ethical and moral decision-making, moral emotions (e.g., guilt, shame, and anger), professional and organizational morals and values, sanctuary trauma, complex grief, and spirituality. Inclusion criteria were: (1) the study population included active or inactive firefighters, paramedics, or police officers; (2) the main topic included moral injury, moral ambiguity, moral or ethical decision making, moral stress, moral distress, values, organizational betrayal, and/or spirituality; (3) the article was accessible in English; (4) the study was peer reviewed; and (5) the article described a research study (i.e., commentaries, theoretical papers, and essays were excluded).

### Study Selection

**FIGURE 1:**
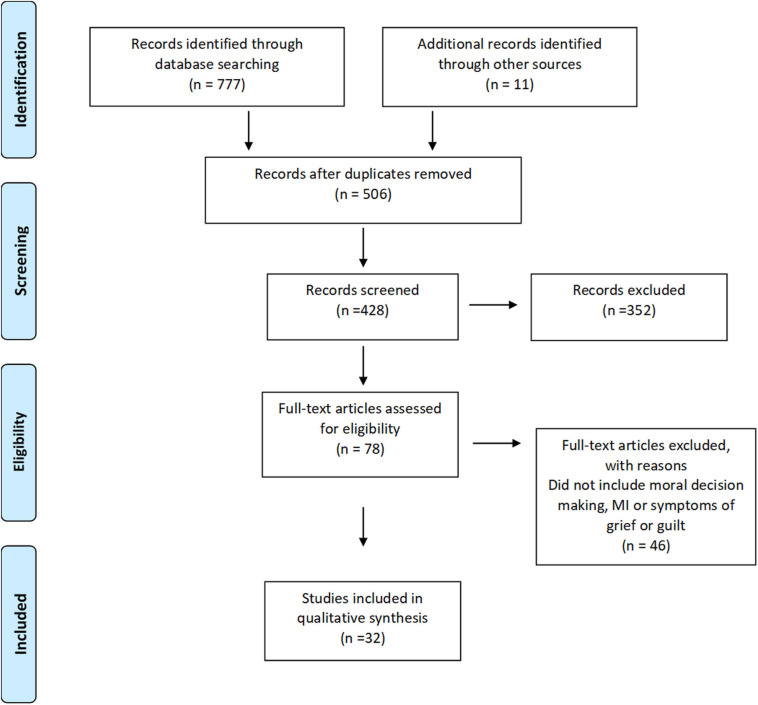
Prisma flow diagram.

The two reviewers independently scanned the titles and abstracts of the articles identified from the initial database search to determine which articles would be selected for further assessment (Arksey and O’Malley, 2005). The inclusion process was iterative and involved refining the search strategy and reviewing articles (Levac et al., 2010). The two reviewers discussed study inclusion and exclusion during an initial conversation to discuss moral injury and associated concepts which may be relevant to include. A hand search of reviewed articles was conducted in order to identify any additional articles of interest. Unanimous agreement was met in regard to which articles to include in the current review (Figure 1).

### Data Charting

The two reviewers jointly developed the data extraction form for collecting relevant aspects of the evidence: author, location, date population, study design, concept being studied, purpose, and results. After the first several articles were read and independently reviewed, the two reviewers used the data extraction form to communicate any required modifications (Levac et al., 2010). The information from relevant articles was extracted and entered into a Microsoft Word 2016 (Windows) table for analysis. The information was narratively analyzed and synthesized (Popay et al., 2006) to identify and summarize the state of the literature on moral and ethical challenges for firefighters, paramedics, and police officers.

## Results

### Included Studies

The initial database searches returned 777 articles and 506 remained after duplicate articles were removed. After screening the title and abstract and assessing the full text, 30 articles were selected for inclusion in the current study (*n* = 32; Figure 1). Police officers were the primary population of interest in 24 studies, paramedics in seven studies and, firefighters in one study. Two studies included mixed populations; one included study involved police officers, firefighters and other emergency service workers, and one other included study that involved both paramedic and firefighter incident commanders. Most included studies were qualitative in nature (*n* = 19) with data gathered primarily from structured or semi-structured interviews. Data from one study was gathered from an in-service discussion and, fieldwork observation was done in two studies. Additionally, one study used both fieldwork observation and interviews whereas four studies used surveys or questionnaires. The studies included covered the following four topic areas related to morally injurious experiences confronted by firefighters, paramedics, and police officers; values, ethical decision-making, organizational betrayal, and spirituality (Table 1).

**TABLE 1:** Included studies.

Author, Year	Country	Topic	Population	Study Design	Research Purpose
Beek and Göpfert, 2013	Ghana and Niger	Ethical decision-making	Police Officers	Qualitative	To explore difficulties that state police personnel face when they use violence.
Brown et al., 2016	United States and Jamaica	Ethical decision-making	Police Officers	Quantitative	To make sense of the relationship between avoidant decision-making and decisions to avoid, or not, within the context of police work.
Chopko and Schwartz, 2012	United States	Spirituality	Police Officers	Quantitative	To investigate the relationship between the degree of posttraumatic distress, exposure to potential work-traumas, and personal effort toward relational and spiritual growth.
Chopko et al., 2016	United States	Spirituality	Police Officers	Quantitative	To what extent spiritual effort and spiritual growth are associated with stress and health symptoms among law enforcement officers.
Dick, 2005	England	Ethical decision-making	Police Officers	Qualitative	To identify how women and men experienced police culture during their day to day activities as police officers through an exploration of how police culture could be characterized and how it affected the lived experiences of policemen and policewomen.
Feemster, 2007	United States	Spirituality	Police Officers	Quantitative	To explore the impact of law enforcement on aspects of police officers’ s spirituality and how spirituality influenced their practice and performance of law enforcement.
Francis et al., 2018	England	Ethical decision-making	Firefighters, Paramedics and Paramedic Students	Quantitative	To investigate simulated moral actions in virtual reality made by professionally trained paramedics and fire service incident commanders who are frequently faced with and must respond to moral dilemmas.
Charles, 2006	United States	Spirituality	Police Officers	Qualitative	To explore how a police officer’s spirituality affects police work and a police officer’s ability to reflect and manage the trauma incurred in their work.
Charles et al., 2014	United States	Spirituality	Police Officers	Quantitative	To explore the relationship between self-reported spirituality and brain patterns of police officers who had previously been interviewed on their spirituality, and how it affected their work as law enforcement officers
Goldschmidt and Anonymous, 2008	United States	Values	Police Officers	Qualitative	To examine police officer attitudes toward and motivations for the use of dishonesty and extralegal means in furtherance of law enforcement function.
Herbert, 1996	United States	Ethical decision-making	Police Officers	Qualitative	To develop an explanation for the prevalence of police morality by illustrating the centrality of morality to everyday understandings and justifications of police actions.
Hesketh et al., 2014	United Kingdom	Spirituality	Police Officers	Quantitative	To explore the relationship between spirituality and resilience, how spirituality is an important aspect of both organizational and personal values, how these values can support resilience programs aimed at improving wellbeing in police organizations.
Hyllengren et al., 2016	Sweden and Norway	Ethical decision-making	Police officers	Qualitative	To identify and gain a deeper understanding of environmental, organizational, and group conditions, and leadership-related issues in severely stressful situations involving moral stressors faced by military and police officers.
Jafari et al., 2019	Iran	Ethical decision-making	Paramedics	Qualitative	To explore EMS staff’s experiences of the factors behind their moral distress.
Janas, 2013	United States	Spirituality	Firefighters	Qualitative	To explore if firefighters who have religious and/or spiritual beliefs report fewer symptoms of trauma and if spirituality is a protective factor.
Jonsson and Segesten, 2004	Sweden	Ethical decision-making	Paramedics	Qualitative	To uncover and deepen the understanding of the way ambulance staff experience and handle traumatic events and to develop an understanding of the life world of the participants,
Kleinig, 1993	United States	Ethical decision-making	Police Officers	Qualitative	Consideration of three conceptions of the police role; law enforcement, order maintenance, and social peacekeeping and indicate how they impinge on treatment of the homeless.
McCormack and Riley, 2016	Australia	Organizational betrayal	Police Officers	Qualitative	To explore the positive and negative subjective “lived” experience of long-term policing and subsequent psychological distress leading to medical discharge with a diagnosis of PTSD. To explore participants’ lived experiences of work-related discharge with a mental health diagnosis; their perception of organizational support for reintegration into civilian life; and whether psychological growth is possible in the aftermath of these events.
Nelson et al., 2020	England	Ethical decision-making	Paramedics	Qualitative	To explore the perspective of ambulance staff responding to deaths by suicide.
Norberg, 2013	United States	Ethical decision-making	Police Officers	Qualitative	To examine how individual values might influence professional values. It stresses the need for reflection concerning the ethical dimension of policing.
Oginska-Bulik, 2013	Poland	Spirituality	Police Officers, Firefighters, and emergency service workers	Quantitative	To investigate the role of personal (spirituality) and social (social support in the workplace) resources in both negative (posttraumatic stress disorder) and positive (posttraumatic growth) effects of experienced trauma in a group of emergency service workers.
Ozcan et al., 2013	Turkey	Ethical decision-making	Paramedics	Quantitative	To assess the ethical issues that emergency care providers have encountered and examine their ethical reasoning in resolving these complex issues.
Patton, 1999	United States	Spirituality	Police Officers	Qualitative	To explore how a police officer’s spirituality is affected by continuous exposure to crime, danger, suffering and violence and whether a state of spiritual wellness assists veteran police officers in coping with the stress in their lives. To identify what interventions are suggested by analysis of the data to provide a holistic approach to counseling with police officers.
Qashu Lim, 2017	United States	Values	Paramedics	Qualitative	To investigate how the work of paramedics, nurses and physicians, within their professional practice spheres of emergency medicine, constantly resolve challenges that make their moral agency visible.
Raganella and White, 2004	United States	Values	Police Officers	Quantitative	To examine motivations for entering police work among New York City Police Department recruits. To explore gender and race differences in motivation.
Reynolds and Helfers, 2018	United States	Organizational betrayal	Police Officers	Qualitative	To address how police officers (a) feel about and (b) respond to perceptions of injustice occurring within their departments.
Smith, 2009	United Kingdom	Spirituality	Police Officers	Qualitative	To investigate if the spiritual dimension of law enforcement should be included in the United Kingdom police force training – the Trainers’ Development Program.
Torabi et al., 2018	Iran	Ethical decision-making	Paramedics	Qualitative	To identify and describe the experiences of EMS personnel in ethical decision making (EDM) when they are faced with ethical dilemmas. To identify and describe the experiences of Iranian pre-hospital emergency service personnel in the field of EDM.
Tovar, 2011	United States	Spirituality	Police Officers	Qualitative	To examine how law enforcement officers reconcile vicarious trauma experiences or disruptions to their core beliefs and manage the physical, psychological, social, and spiritual ramifications.
Westmarland, 2005	England	Values	Police Officers	Quantitative	To analyze evidence from a survey of police officers who were asked about their attitudes toward police corruption, unethical behavior and minor infringements of police rules
White et al., 2010	United States	Values	Police Officers	Quantitative	To examine the stability of motivations for becoming police officers and to examine the relationship between these motivations and job satisfaction. This is a continuation of Raganella (2004)
Zhao et al., 1998	United States	Values	Police Officers	Quantitative	To make use of the theory of human values developed by Rokeach as a theoretical framework to evaluate value preferences among sworn officers in American police agencies. To investigate three issues: (1) What are the value orientations of police officers today? (2) Have such value orientations among police officers changed over time? (3) Is there a consensus on values among officers, or does considerable diversity of values obtain across subpopulations within the law enforcement workforce?

### Values

Values have been defined as “a concept of the desirable with a motivating force” (Hodgkinson, 1991, p. 101). In other words, values are that which is good, desirable, or worthwhile and motivate purposeful action. There were six studies that investigated value orientations held by PSP (Zhao et al., 1998; Raganella and White, 2004; Westmarland, 2005; Goldschmidt and Anonymous, 2008; White et al., 2010; Qashu Lim, 2017). The value orientations of police officers were assessed in five of the six studies. The single study looking at paramedics’ values indicated that paramedics are motivated by a desire to help their patients and community to be better (Qashu Lim, 2017). The desire to help people appears as a transcendent theme driven by a desire to help others and work toward a greater good. Values held by police officers can be deduced from a study done in 2002 where the main motivation for recruits to join the New York Police Department (NYPD) was the opportunity to help while power and authority ranked lowest (Raganella and White, 2004). In 2008, members of the NYPD indicated that the opportunity to help remained a strong motivating factor for remaining a police officer while power and authority continued to remain irrelevant (White et al., 2010). Police officer values appear stable over time. In a study spanning the 1970s to 1998 the value items ranked as most important for police officers were family security, happiness, self-respect, freedom, true friendship and inner harmony, whereas social recognition, pleasure, and an exciting life ranked of lower importance (Zhao et al., 1998).

Dishonesty as a ‘value’ is likely viewed as an unethical practice in general society; however, police officers occasionally use dishonesty in their work to achieve an outcome that benefits the greater good. Goldschmidt and Anonymous (2008) found that the use of dishonesty in police work is motivated by working for the greater good; however, a clear sliding scale of morality was identified. For example, falsifying reports was seen as acceptable when necessary to protect against failures of the criminal justice system, despite reservations held by police officers. Conversely, planting evidence was viewed as a much more serious ethical violation. The acceptance of dishonesty varied according to the seriousness of the crime where the more serious the crime, the greater level of dishonesty was acceptable as long as there was minimal risk of the dishonest act causing the court case to be lost (Goldschmidt and Anonymous, 2008). Westmarland (2005) took a more general look at police ethics and also found a continuum of professional ethics among police officers. Survey results indicated that police officers were quite discriminating on what they thought were most and least serious violations of the morals in police culture. Specifically, behavior that involved acquiring money or property for personal gain was deemed most serious regardless of the monetary value associated with it (Westmarland, 2005).

### Ethical Decision-Making

A four-component model of ethical decision-making and behavior was suggested by Jones (1991): the moral agent (1) recognizes the moral issue; (2) makes a moral judgment; (3) resolves to place moral concerns ahead of other concerns (establish moral intent); and (4) act on the moral concerns. For PSP, ethical decisions are based on the moral issue of who will benefit from one’s actions and who may be harmed (Norberg, 2013). PSP must, quickly and in a complex situation, consider the risk-benefit of their actions and do what they feel will benefit the member of the public or the community prior to taking action. Several articles focused on the complexity of tasks, the ambiguous circumstances, and the conflicting roles that PSP encounter daily.

As they navigate between being agents of the government enforcing laws, acting as social peacekeepers and, mediating of communal welfare, police fulfill a complex position in society (Kleinig, 1993). It can be uncomfortable for police when they must deal with individuals in a public health context, for example, treating the homeless with compassion, while facing pressure of having to do something (i.e., remove the homeless person from a location that they are not wanted) to satisfy whomever sees the homeless as a public nuisance (Kleinig, 1993). Police officers are confronted daily with the task of having to make decisions on how to act, often quickly, to resolve criminal incidents, perhaps with the use of physical or lethal force, and this can cause a clash between legislation and morality (Norberg, 2013) or the personal and professional (Dick, 2005). Police actions are intended to protect good through elimination of evil, which is arguably a futile task (Herbert, 1996). As such, in many situations police officers face an inherent ambiguity. Officers must make rapid decisions on the credibility of both victims and complainants by weeding through different accounts of an event quickly, decisively, and hopefully fairly. Officers may need to act against at least one person’s interest in most situations and potentially use physical force to accomplish their task (Herbert, 1996). Intuitively the desire to help clashes with the necessity of harm. Policing is one of the few circumstances, next to military work, where use of force is a necessary and accepted tool that increases moral complexity.

In the course of their duties police officers may be required to use force and act outside of their normal tendencies (Brown et al., 2016). Police officers will avoid using violence by attempting to calm down agitated or belligerent civilians to minimize moral struggle (Beek and Göpfert, 2013); however, when police officers must take an enforcement approach (e.g., apprehend a resistive or combatant suspect) they may be unable to opt out of the morally difficult decision to use violence (Norberg, 2013). As police officers progress through their career some may develop the ability to separate their personal life from the professional life by becoming a part or agent of the organization (Dick, 2005). By viewing use of force in a broader scheme of the organization, state or, society rather than as an individual officer the moral ambiguity of using force is decreased. Police officer conduct at work becomes part of a role rather than one’s person (Dick, 2005). Additionally, using force is seen as a result of prisoner or suspect actions rather than a personal choice made by the officer (Herbert, 1996).

Paramedics are also confronted with conflicting roles. At an emergency scene, for example, a paramedic must not only deal with their patient, they must also be conscious of possible dangers to themselves, bystanders or, family at the scene. Paramedics may have to deny family access to their loved ones either to protect a crime scene or to protect the family from seeing their loved one in pain or an unimaginable circumstance (Nelson et al., 2020). Additionally, paramedics are the experts at an emergency medical call, but upon arriving at the emergency department they can feel devalued and have a sense of not being heard or listened to by emergency room personnel (Qashu Lim, 2017; Nelson et al., 2020).

Like police officers (Hyllengren et al., 2016), paramedics’ moral agency is often challenged when they are faced with situations without authority and/or the tools to act (i.e., not having the authority to provide a certain painkiller while simultaneously knowing the patient is in extreme pain) (Qashu Lim, 2017). Several organizational factors have been identified that may be related to paramedics’ moral distress in regard to decision-making and the ability to take action (Jafari et al., 2019). Lack of resources and restraints on care provision were identified, but other factors that may lead to moral distress included coworkers’ lack of knowledge and competence, ineffective communication, and differences in values which resulted in conflicts regarding what and how occupational tasks should be completed (Raganella and White, 2004). Performing artificial services or interventions in order to avoid complaints or to satisfy the expectations of bystanders was associated with increased moral distress in pre-hospital care personnel (Bremer and Sandman, 2011). The impact of performing unnecessary artificial intervention (i.e., performing CPR on a patient who will clearly not benefit) on paramedics may be related to their strong commitment to help and heal. Interviews with Swedish ambulance personnel identified that the fear of failing in their responsibility to take care of patients, and feelings of not doing enough for patients, evoked feelings of insufficiency and worthlessness (Zhao et al., 1998; Jonsson and Segesten, 2004). Additionally, feelings of disrespect and demoralization come from the treatment they receive from colleagues in other medical disciplines (Goldschmidt and Anonymous, 2008).

Paramedics also consider several individually based factors in their ethical decision-making process. Patient values, confidentiality, opinions, and beliefs are of utmost importance to paramedics (Westmarland, 2005). Not adhering to a patient’s values causes distress to paramedics. Additionally, ethical dilemmas arise from a conflict between truth telling and protecting the wellbeing of family members when paramedics are faced with deciding to share bad news to family members or perhaps lie to them about the gravity of a patient’s condition in an attempt to protect loved ones from psychological pain or distress (Norberg, 2013; Ozcan et al., 2013). By maintaining a professional commitment and performing tasks within the framework outlined by the regulations under which they must work, paramedics are able to partially alleviate the ethical dilemmas and moral distress associated with their job tasks (Norberg, 2013). Research indicates that, personal values and beliefs, along with professional experiences gained from working with expert colleagues, was the greatest factor that facilitated ethical decision making in pre-hospital emergency personnel (Torabi et al., 2018).

Moral judgments and moral actions do not appear to differ between civilians and professionally trained paramedic and fire service incident commanders; however, paramedic and fire service incident commanders appear to have an increased physiological resilience when confronted with a moral action task (Francis et al., 2018). When heart rates were compared between civilians and incident commanders faced with completing non-moral and moral action and judgment tasks, civilian heart rates increased while heart rates decreased for incident commanders. Additionally, after task completion, civilians expressed feelings of regret at taking what they felt was the wrong action whereas the incident commanders were more confident and felt they took correct action (Francis et al., 2018). The mechanism for the difference in physiological response, whether a personality trait or a result of training and experience, is unclear.

### Organizational Betrayal

Broadly defined, organizational betrayal is a description of individual experiences of violations of trust and dependency perpetrated against any member of an institution, or when an institution causes harm to an individual who trusts or depends upon that institution (Smith and Freyd, 2014). Two qualitative studies examined former and current police officers’ feelings regarding organizational injustice and betrayal (McCormack and Riley, 2016; Reynolds and Helfers, 2018). When asked of incidents where American police officers felt that they were not treated fairly by their organization, four main events were identified: disciplinary action, administrative resolution of citizen complaints, supervisor altercations, and blocked career aspirations (Reynolds and Helfers, 2018). Most often, police officers stated that reactions to these events elicited feelings of anger (75%) followed by feelings of unappreciation, frustration, and disappointment. As a result of these experienced and associated feelings, officers became more skeptical of their organization and they perceived that their organization did not support them. In turn, the changes in attitude had negative effects on the officers’ willingness to put effort into the organization and their work while putting an increased effort in to self-preservation within the organization (Reynolds and Helfers, 2018).

Police officers have also felt betrayed by their organization after having left. McCormack and Riley (2016) explored the lived experiences of police officers who suffered PTSD because of the trauma and distress experienced during their policing careers. Participants in this study were all medically discharged from their duties as police personnel in an Australian police service due to PTSD. The authors argue that participants’ continuing PTSD symptoms and resistance to intervention may be due to moral injury and that continued anguish and distress prohibits opportunities for growth and wellbeing in this group. Participants interpreted their discharge from the police service due to a mental health diagnosis as a lack of support from the organization leading to feelings of shame, failure, and an eroded self-worth. They found themselves disconnected without support for acclimatizing to civilian life while no longer being a part of the police culture. A sense of invalidation ensued along with feelings of being dispensable as betrayal began to overshadow the on-duty exposures that may have lead to PTSD. Attempts at open and honest communication within the police service were met with negativity; however, after discharge, some participants began to see the benefits of honest communication outside of the service which led to hope and a new appreciation of self (McCormack and Riley, 2016).

### Spirituality

Spirituality understood as – beliefs, values, behaviors and experiences that give meaning and purpose to a person’s life and connectedness to the significant or sacred (Brémault-Phillips et al., 2019) – has been identified as being relevant to the discussion of moral injury. Eleven studies focused on the construct of spirituality and how that impacted the psychological spiritual domains of health, nine of which focused on spirituality in police officers, one on firefighters, and one examined all three PSP groups. Results from the studies were mixed regarding the potential importance and impact of spirituality on psychological health. Three articles looked at if spirituality was a protective factor against exposure to traumatization and subsequent PTSD symptoms and showed that spirituality was not protective (Chopko and Schwartz, 2012; Janas, 2013; Chopko et al., 2016). Chopko et al. (2016), but demonstrated that while spirituality was not protective overall, there was a positive association between spiritual growth and psychological distress, and that spirituality was inversely related to alcohol use in police officers. Similarly, Janas (2013) showed that an increased number of traumatic events was a predictor of stronger spiritual beliefs indicating firefighters may have experienced posttraumatic growth. Oginska-Bulik (2013) determined that two dimensions of spirituality–harmony and religiousness–in addition to coworker support turned out to be the strongest predictors of posttraumatic growth in police, firefighters, and paramedics exposed to trauma. Additionally, police officers with a greater sense of spirituality may have better coping mechanisms and self-actualization than those with a lower sense of spirituality (Charles et al., 2014).

The remaining six articles explored the construct of spirituality within police work. There were three studies (Charles, 2006; Smith, 2009; Hesketh et al., 2014) that noted spiritual elements were often foundational to police work (e.g., seeing being a police officer as a vocation/calling, the desire to serve the greater good or help people, the ability to protect or provide compassionate care to those who have been harmed, finding meaning and purpose, and bearing witness to experiences of human destructiveness). Integration of spirituality in police training may support officers to self-actualize, increase their effectiveness of managing their emotions, and reduce some of the negative implications of police organizations and culture (Smith, 2009). It has been noted that in addition to traditional trauma symptoms (i.e., hypervigilance, avoidance, somatic complaints) officers also experienced struggles with spiritual erosion (i.e., loss of relationship with a higher power or engagement in spiritual practices), negative changes in worldviews (i.e., bitterness, cynicism, jadedness), and a loss of sense of meaning (Tovar, 2011). Similarly, Patton (1999) noted that police officers experienced pronounced spiritual pain and distress resulting in disappointment, disillusionment, loss of self, meaninglessness, desacralization, alienation, hopelessness, and existential questioning. Finally, Feemster (2007) illustrated that the problem of “evil” was central to policing with 58% of the 747 respondents sampled stating they had encountered evil in forms for which 70% of participants felt inadequately prepared to encounter.

## Discussion

The current scoping review explored moral injury, values, moral dilemmas, and moral decision-making as regularly faced by firefighters, paramedics, and police officers and identified a limited number of studies on the topic. Most notably, no research articles exploring moral injury in PSP were identified. This dearth of evidence illustrates that while it is arguable that moral injury is likely to have relevance within PSP work, current understanding of what moral injury is and how it may relate to PSP occupations and service environments is lacking. Within military literature, rightful criticism has occurred regarding the lack of standardized operational definition and poor ontological conceptualization of moral injury (Griffin et al., 2019; Litz and Kerig, 2019). This challenge will be intensified in the public safetycontext as future research will need to ensure that moral injury is properly and adequately understood in light of the specific contextual occupational factors of each public safety service environment. For example, in the current review only three studies explored these concepts in firefighters (Brough, 2004; Janas, 2013; Oginska-Bulik, 2013). This is troubling as firefighters are almost twice as likely as the average Canadian adult to consider suicide (Statistics Canada, 2015; Carleton et al., 2018) and are more commonly exposed to human suffering and death (Carleton et al., 2019) –a known PMIE (Vargas et al., 2013; Schorr et al., 2018).

The articles included in the current review indicate that PSP are strongly guided by personal values yet are often exposed to complex moral dilemmas that incite moral distress when they have to make difficult ethical decisions. Arguments have been put forward that PSP act according to personal norms and discretion rather than the norms dictated by organizational policies, which do not deal with the urgency and danger inherent in the situations PSP face (Boin and Nieuwenburg, 2013). In an emergency context, PSP need to act quickly and often improvise as they balance the action that is most protective or helpful while being true to their personal, professional, and societal moral norms (Boin and Nieuwenburg, 2013). Reay et al. (2018) noted in their research about paramedic decision-making that current prescribed protocols were too static to be meaningfully applied to the fluid and dynamic nature of pre-hospital care environments, resulting in paramedics frequently using their own clinical judgments.

In professions where there is a hierarchical structure, as in many public safety occupations, individuals may struggle with locus of control (LOC). Given the rank structure, PSP may feel obligated to act on superior officer commands or strictly within rules of engagement or operational policy. This perpetuates a LOC that is external to the individual. External LOC has been indicated as a risk factor for PTSD and correlate of psychopathological symptoms. Conversely, an internal LOC, has been shown to be a protective factor (Schäfer et al., 2020). Being influenced by an external LOC may interfere with moral agency and further exacerbate ethical dilemmas (Norberg, 2013; Qashu Lim, 2017). For example, in the above paramedic context, while a paramedic may frequently desire to use their clinical judgments they are still legally, professionally, and ethically obligated to use pre-established protocols. This tension may be intensified if a paramedic feels that following the protocol (i.e., the external LOC) may cause undue harm to a patient versus following their own clinical judgment (i.e., internal LOC). Similarly, Currier et al. (2015) found that in order to preserve their moral dignity and agency, soldiers may choose to act according to their own moral code, rather than follow organizational rules. While acting according to one’s personal moral code could reduce the likelihood of sustaining moral injury, it may lead to disciplinary action as the person acted against organizational standards or did not wait for orders before they acted. It is important then, that organizational leaders also demonstrate discretion and understanding when PSP act out of line with organizational standards particularly when they are faced with complex moral dilemmas.

Moral complexity can also arise from the conflicting roles that PSP play while enacting their duties and the desire to help, not harm. This is especially true for police officers, who are tasked with being able to use lethal force, while they are the most visible agents of the justice and health systems. This leaves them open to public criticism and increased pressure to conform to different demands from a variety of sources (Cebulak, 2001). The traumatic experiences of PSP are complicated by citizen complaints, media attention, and internal or external criminal investigations all of which add to the stress of their work (Komarovskaya et al., 2011). Cebulak (2001) argued that police officers are disadvantaged by the public’s expectation that they must be fair in their actions to protect the public from those who are unfair and unjust in their criminal actions; this arguably could foster the development of moral injury. Within current climates of public distrust in some PSP groups, this tension between PSP individual personal morals and authenticity may be significantly challenged when they must also be seen to publicly uphold one’s professional duty and organizational morals and values.

To support frontline personnel when they are faced with a moral dilemma, all of these considerations, and more, need to be taken into account preventatively through ethics and moral training. However, careful consideration of how this ethics and moral training is offered and what to include in it, is paramount. While ethics training have been implemented within Western military contexts, it has been found to be ineffective because of the focus on theoretical and legalistic issues, and the descriptive academic approach of teaching rules and regulations, coupled with an enormous amount of informal, indirect, unsystematic education (Robinson et al., 2008; Baarle et al., 2015). This point was vividly illustrated by the Mental Health Assessment Team that examined the wellbeing of US soldiers deployed in Iraq and found that issues of unethical behaviors were frequent despite military members having taken ethics training (Castro and McGurk, 2007). For example, more than 28% of soldiers and 31% of marines reported facing ethical situations to which they did not know how to respond (Castro and McGurk, 2007). Moreover, most soldiers deployed to Iraq were unsatisfied with traditional PowerPoint presentation approaches to operational ethics preparation, which they felt did not adequately reflect the realities of their combat experiences (Warner et al., 2011).

In response, high fidelity ethical scenarios have been suggested as a potential means of conducting ethics training for military personnel both pre and within deployment (Wortel and Bosch, 2011; Thompson and Jetly, 2014). Central to this type of teaching approach is the idea that all military ethics-training must include components which increase moral awareness, confidence, and mastery, along with the ability to practice moral agency and judgment (Johnson, 2011). Some authors have suggested that this type of training is more akin to “moral resiliency training” than traditional ethics training. Moral resilience–the capacity of an individual to sustain or restore their integrity in response to moral complexity, confusion, distress, or setbacks–has been proposed in healthcare literature as a preventative approach to address moral injury and distress (Rushton, 2017). Similar to other forms of resilience, proponents of moral resilience have argued that this skillset cultivated through knowledge, atonement, sensitivity, reflection, agency, and practice, creates healthcare workers who are more morally competent, integrated, efficacious, and effective (Young and Rushton, 2017). While the use of high-fidelity ethics training to support moral resilience in PSP is potentially promising, the efficacy of any of these approaches have not been studied, and therefore caution is warranted.

Having a sense of control (discretion and agency) and preparedness (training) along with understanding one’s values can decrease the potential of psychological injury and moral injury but may not decrease moral distress (Papazoglou and Chopko, 2017). Instead, it may be equally important to prepare PSP to manage their emotional, cognitive, existential, and spiritual distress when faced with a moral dilemma which is unresolvable, or when they have experienced moral injury. Borrowing from the posttraumatic growth literature, it may be that the most effective manner in which to reduce moral distress and moral injury is to provide specific mental, emotional and spiritual tools to address intense emotions, resolve internal dissonance, integrate fractured belief systems, rebuild trust and social connections, and engage in forgiveness and compassion practices (Worthington and Langberg, 2012; Bryan et al., 2015; Smith-MacDonald et al., 2018; Kelley et al., 2019). Greater attention to mental health services after PMIE can also serve as a preventative measure for PSP who have been negatively impacted by their exposure to PMIE (Komarovskaya et al., 2011).

The perception of organizational support may allow employees to feel valued (Eisenberger et al., 1986); however, the disillusionment of this perception–when the organization cannot live up to the perception–can have negative consequences. Smith and Freyd (2014) note that organizational betrayal is associated with complex mental health outcomes similar to those associated with interpersonal betrayal and trauma. This organizational betrayal seems to be rooted in personal perceived bureaucratic attributes of the organization and feelings that organizations fail to support their human resources or where leadership appear to be incompetent or uncaring. Research has identified that both organizational and occupational components contribute to psychological strain and decreased work satisfaction when transgressions by peers, leaders, or organizations that betray moral/ethical beliefs or expectations occur (Brough, 2004; Shay, 2014). This research has also suggested the feelings of betrayal and lack of self-worth were linked to medical discharge for a mental illness. This is likely related to the stigma toward ill mental health in PSP culture and public safety organizations. Consequently, when a police officer suffers from a mental health disorder, this may be seen as a sign of poor character leading to devalued social identity, placing officers in the same category as many of the subjects that they interact with daily (Yang et al., 2007; Bullock and Garland, 2018). This demotion to be the “other” may alter a police officer’s identity in both a personal and social context bringing into question the officer’s ability to perform their duties (Bullock and Garland, 2018).

Stigma may be a moral experience whereby the stigmatized are no longer able to fulfill their role as a moral citizen by meeting social obligations and norms. The stigmatized are also unable to hold on to what matters most to ordinary people in a society (Yang et al., 2007; Kleinman and Hall-Clifford, 2009). In the public safety context, this manifests as the inability to continue working in full capacity and either being medically discharged or placed in a modified work environment (Bullock and Garland, 2018). Other moral experiences where PSP can feel as though they are treated as the other or as inferior compared to their counterparts include unbalanced disciplinary action, administrative resolution of citizen complaints, supervisor altercations, and blocked career aspirations. Perceived rejection by the organization may decrease one’s sense of belonging, a fundamental human need (Baumeister and Leary, 1995; Gere and MacDonald, 2010), and lead to depression and stigmatization along with the associated moral experiences (Hagerty et al., 1996; Hagerty and Williams, 1999; Yang et al., 2007; Bullock and Garland, 2018). A reduction in mental health stigma may decrease the impact mental ill health may have on PSP within their organization and community. A reduction in stigma and an openness toward discussion and acceptance that mental health disorders are common in PSP, and are most likely temporary, may decrease moral distress in this community and increase help-seeking.

Finally, the issue of spirituality has great relevance to the discussion of moral distress and moral injury but is also challenging to research. As the construct of moral injury continues to be explored and developed, spirituality and spiritual distress have been recognized in the literature as core features of moral injury (Carey et al., 2016; Wortmann et al., 2017; Doehring, 2019). Studies of military personnel’s first-hand experiences suggest that spiritual and existential struggles were commonly reported following exposure to PMIEs (Vargas et al., 2013; Purcell et al., 2016; Lancaster and Miller, 2019). Currier et al. (2019) has proposed that moral injury may consist of two subtypes – psychological or spiritual/religious–which share commonalities but have specific care needs. However, as our results showed, understanding how spirituality impacts both moral injury and subsequent mental health challenges is complex and nuanced. Similar to moral injury research, spirituality research has been noted to be problematic because of the lack of standardized operational definition, the multidimensional aspect of the construct, and poor reliability and validity in many of the psychometric instruments used (Zinnbauer et al., 1999; Koenig, 2008; Smith-MacDonald et al., 2017).

A growing body of literature is noting that in relation to health it is important to determine if a person is experiencing positive or negative spirituality. In a systematic review, Smith-MacDonald et al. (2017) found that negative spiritual coping (e.g., alienation from one’s higher power, sense of hopelessness or meaninglessness, fractured belief systems) was strongly correlated with increased mental illness (e.g., PTSD, depression, anxiety), suicidal ideation, and poor quality of life with veterans, while positive spiritual coping was found to have the opposite influence. Similar results were found in a review by Brémault-Phillips et al. (2019) on moral injury and spirituality in veterans. Consequently, PSP may be able to use positive spiritual coping may experience elements of posttraumatic growth, while PSP who use negative spiritual coping may be inadvertently perpetuating harmful elements of exposure potentially psychologically traumatic events. A person may have aspects of both negative and positive spiritual coping occurring simultaneously (e.g., attempting to use spiritual or religious practices as a positive means of coping but are also experiencing anger at their higher power) which may be a crucial component for future research to consider. Finally, theodicy–the question of good and evil – needs to be more thoroughly addressed. The cross-sectional results from the Feemster (2007) study with American police officers illustrate that questions of good and evil are prevalent in police and potentially in other PSP populations. No research has properly explored the current issues, or to provide solutions or recommendations regarding how PSP make sense and emotionally cope with issues of theodicy.

## Conclusion

Public safety personnel function in an environment where duty, care, and moral agency intersect with human tragedy. The intersections facilitate the very best and the very worst in individuals as a function of their preparedness to carry out their professional duty, to care for those they have sworn to protect, and to do so in a manner that is consistent with their personal values. The current review identified four dominant themes related to the moral toll (injury and distress) PSP may experience in their daily efforts to keep the public safe: values, ethical decision-making, organizational betrayal, and spirituality. Themes and the limited amount of available research contained within them are somewhat disparate, but there is common ground for public safety organizations being able and willing to address moral injury and moral distress. The current results identified the centrality of personal values relative to formal and informal organizational values, duty, and expectations in the moral landscape of working in public safety. Moral injury and distress are the result of the disconnect between what the PSP is asked to do or witness and what is a core personal value–the essence of the individual. Organizational best efforts are often designed to foster institutional values, codes, and duties, but may neglect and expense individual self-awareness. The limited data that does exist for PSP identifies many opportunities to better understand moral injury and moral distress, that military data is insufficient to explain moral injury among PSP, and that moral preparedness training must be directed toward implicit individual value awareness before PSP confront potentially psychologically traumatic events.

## Author Contributions

LL and LS-M drafted this manuscript. LL, LS-M, DM, RC, and SB-P revised this draft. All authors reviewed and approved this manuscript.

## Conflict of Interest

The authors declare that the research was conducted in the absence of any commercial or financial relationships that could be construed as a potential conflict of interest.
