# Supplementary material for: Compromised Conscience: A Scoping Review of Moral Injury Among Firefighters, Paramedics, and Police Officers
Source: Front Psychol. 2021 Mar 31;12:639781. doi: 10.3389/fpsyg.2021.639781 (PMC8044342; doi:10.3389/fpsyg.2021.639781)
Supplement: Supplementary file 1 [file Data_Sheet_1.docx]

**Appendix 1**

**Scoping Review Search Terms**

| **Database** | **Search Terms** |
| --- | --- |
| *CINAHL PLUS* | S1: (MH "Spiritual Distress (NANDA)") OR ( ((moral* N4 (injur* or repair or dilemma* or distress* or conflict* or difficult* or challeng*)) or "sanctuary trauma" or disenfranchised grief or complex grief) ) OR ( (spiritual* N8 (wellbeing or well being or wellness or struggl* or trauma* or conflict* or distress*)) or ((organizational or institutional) N4 (betrayal or trauma*))) ) OR ( ((guilt or shame or grief or betrayal or anger or angry or regret*) N12 (moral* or event* or action* or trauma* or incident* or situation or situations)) )  S2: ( (MH "Firefighters") OR (MH "Police") OR (MH "Emergency Medical Technicians") ) OR ( (police officer* or policem* or policewom* or police personnel or police professional* or firefighter* or firem* or EMT or EMTs or EMS or (Emergency Medical N2 (personnel or technician*)) or paramedic* or "public safety personnel" or public safety professional* or "first responder*" or "law enforcement" or "medic" or medics or ambulance) )  S3: S1 AND S2 |
| *SCOPUS* | ( TITLE-ABS-KEY ( ( moral* W/4 ( injur* OR repair OR dilemma* OR distress* OR conflict* OR difficult* OR challeng* ) ) OR sanctuary-trauma OR disenfranchised-grief OR complex-grief ) OR TITLE-ABS-KEY ( spiritual* W/8 ( wellbeing OR well-being OR wellness OR struggl* OR trauma* OR conflict* OR distress* ) ) OR TITLE-ABS-KEY ( ( organizational OR institutional ) W/4 ( betrayal OR trauma* ) ) OR TITLE-ABS-KEY ( ( guilt OR shame OR grief OR betrayal OR anger OR angry OR regret* ) W/12 ( moral* OR event* OR action* OR trauma* OR incident* OR situation OR situations ) ) ) AND ( TITLE-ABS-KEY ( police-officer* OR policem* OR policewom* OR police-personnel OR police-professional* OR firefighter* OR firem* OR emt OR emts OR ems OR ( emergency-medical W/2 ( personnel OR technician* ) ) OR paramedic* OR "public safety personnel" OR public-safety-professional* OR first-responder* OR law-enforcement OR medic OR medics OR ambulance ) ) |
| *Web of Science* | #1 TS=((moral* NEAR/4 (injur* or repair or dilemma* or distress* or conflict* or difficult* or challeng*)) or sanctuary-trauma or disenfranchised-grief or complex-grief) OR TS=(spiritual* NEAR/8 (wellbeing or well-being or wellness or struggl* or trauma* or conflict* or distress*)) OR TS=((organizational or institutional) NEAR/4 (betrayal or trauma*)) OR TS=((guilt or shame or grief or betrayal or anger or angry or regret*) NEAR/12 (moral* or event* or action* or trauma* or incident* or situation or situations))  #2 TS=(police-officer* or policem* or policewom* or police-personnel or police-professional* or firefighter* or firem* or EMT or EMTs or EMS or (Emergency-Medical NEAR/2 (personnel or technician*)) or paramedic* or "public safety personnel" or public-safety-professional* or first-responder* or law-enforcement or medic or medics or ambulance)  #3 #1 AND #2 |
| *EMBASE* | 1. exp fire fighter/ or police/  2. rescue personnel/  3. ((moral* adj4 (injur* or repair or dilemma* or distress* or conflict* or difficult* or challeng*)) or "sanctuary trauma" or disenfranchised grief or complex grief).mp.  4. ((spiritual* adj8 (wellbeing or well being or wellness or struggl* or trauma* or conflict* or distress*)) or ((organizational or institutional) adj4 (betrayal or trauma*))).mp.  5. ((guilt or shame or grief or betrayal or anger or angry or regret*) adj12 (moral* or event* or action* or trauma* or incident* or situation or situations)).mp.  6. (police officer* or policem* or policewom* or police personnel or police professional* or firefighter* or firem* or EMT or EMTs or EMS or (Emergency Medical adj2 (personnel or technician*)) or paramedic* or "public safety personnel" or public safety professional* or "first responder*" or "law enforcement" or "medic" or medics or ambulance).mp.  7. 3 or 4 or 5  8. 1 or 2 or 6  9. 7 and 8 |
| *Medline* | 1. ((moral* adj4 (injur* or repair or dilemma* or distress* or conflict* or difficult* or challeng*)) or "sanctuary trauma" or disenfranchised grief or complex grief).mp.  2. ((spiritual* adj8 (wellbeing or well being or wellness or struggl* or trauma* or conflict* or distress*)) or ((organizational or institutional) adj4 (betrayal or trauma*))).mp.  3. ((guilt or shame or grief or betrayal or anger or angry or regret*) adj12 (moral* or event* or action* or trauma* or incident* or situation or situations)).mp.  4. (police officer* or policem* or policewom* or police personnel or police professional* or firefighter* or firem* or EMT or EMTs or EMS or (Emergency Medical adj2 (personnel or technician*)) or paramedic* or "public safety personnel" or public safety professional* or "first responder*" or "law enforcement" or "medic" or medics or ambulance).mp.  5. 1 or 2 or 3  6. exp Emergency Responders/  7. 4 or 6  8. 5 and 7 |
| *OVID* | TOPIC: ("moral* injur*" or "moral repair" or "moral dilemma*" or "morals" or "moral distress" or guilt or shame or grief or "compassion fatigue" or betrayal or "sanctuary trauma" or "moral Suffering" or spirit*) *AND* TOPIC: (police* or firefighter* or firem* or EMT or EMTs or Emergency medical technician* or paramedic* or "public safety personnel" or "first responder*" or "law enforcement" or "medic" or medics or ambulance) |
